# Spontaneous rupture of the pulmonary hydatid cyst in a young male leading to a complicated pneumothorax: A case report and review of literature

**DOI:** 10.1016/j.ijscr.2025.112073

**Published:** 2025-10-16

**Authors:** Massoud Baghai Wadji, Vahid Ahmadi, Parnian Soltani, Ali Amirkafi

**Affiliations:** a Department of Surgery, Firoozgar Hospital, Iran University of Medical Sciences, Tehran, Iran; b School of Medicine, Iran University of Medical Sciences, Tehran, Iran

**Keywords:** Hydatic cyst, Cyst rapture, Pleural empyema, Thoracotomy, Surgical management

## Abstract

**Introduction:**

Hydatid disease, caused by the *Echinococcus* tapeworm, can form cysts in various parts of the body, mostly in the liver and lungs. These cysts may remain asymptomatic in the lungs or, upon rupture, can lead to pneumothorax and empyema.

**Case presentation:**

We present a case of a 22-year-old male presented with sudden onset of chest pain and dyspnea. After being referred to our hospital and based on physical examination and CT scan findings, he was diagnosed with spontaneous rupture of a pulmonary hydatid cyst. During thoracotomy, massive amounts of empyema and necrotic tissues were discovered in the pleural cavity which were surgically removed. The patient had unremarkable recovery; as his respiratory symptoms were totally resolved, he was discharged within a week with a chest tube inserted.

**Discussion:**

Despite being rare, hydatid disease should be considered as a differential diagnosis for pneumothorax, pleural effusion and empyema in the pleural cavity, particularly in endemic regions. Non-complicated cysts, could be discovered by chest X ray, However, for complicated cysts, CT scan is the choice for diagnosis.

**Conclusion:**

In endemic regions, hydatid cysts should be considered an important differential diagnosis for nonspecific respiratory symptoms, and a surgical approach should be considered for excising the cyst remnants.

## Highlights

• In the endemic regions of hydatid disease, unspecific pulmonary symptoms should be assessed more carefully, with having hydatid cyst in mind.
• Lungs are the second most common site of cyst formation, following the liver and clinical symptoms vary by the size or condition of the cyst.
• Hydatid cysts may remain asymptomatic in the lungs or upon rupture, result in pneumothorax and empyema.
• In the case of non-complicated cysts, chest X ray is proved to be beneficial; otherwise, for complicated cysts, CT scan is the choice of imaging.
• Surgery is the choice of treatment, particularly in patients with complicated or ruptured cysts.

## 1 Introduction

Hydatid disease, also known as human Echinococcosis, is caused by the *Echinococcus* tapeworm. Among the four pathogenic species, *Echinococcus granulosus* and *Echinococcus multilocularis* are the most prevalent [1].

Humans typically acquire the infection by ingesting food or water contaminated with *Echinococcus* eggs, often through contact with infected dogs or consumption of raw or undercooked meat from intermediate hosts like sheep or cattle. Ingested eggs of this tapeworm, release embryos in the small intestine and after penetrating the Upon ingestion of the tapeworm's eggs, embryos are released in the small intestine. After penetrating the mucosa, the larvae spread throughout the body [2]. The liver is the most common site for cyst formation (in over 65 % of cases), followed by the lungs (25 %), with other organs such as the spleen, kidneys, heart, bones, and even the central nervous system being affected in rarer instances [3].These cysts often remain asymptomatic for years due to the silent nature of the disease and are typically diagnosed incidentally—either through imaging or after the onset of nonspecific symptoms caused by compression of surrounding organs or cyst rupture [3].

Cysts may rupture spontaneously, after trauma, or due to sudden pressure. When a pulmonary cyst ruptures, cytokine release and inflammation can lead to nonspecific symptoms such as sudden cough, fever, hemoptysis, and anaphylactic shock [4]. A ruptured cyst may spill into the pleural cavity or bronchus. If it ruptures into the pleural cavity, pleural effusion, infection, and empyema can occur (in 7.6 % of cases), or other complications such as pleural thickening, lung collapse, and simple pneumothorax may develop (in 2.4 to 6.2 % of cases) [5,6].

In such cases, patients may be misdiagnosed with more common pulmonary diseases and, consequently, may receive inappropriate treatment [6].

Although hydatid disease is a rare condition, it remains endemic in sheep- and cattle-raising communities, including in Iran, where it continues to be a public health concern [7,8]. Here, we present a case of a spontaneously ruptured pulmonary hydatid cyst with rapid onset of empyema, which required thoracotomy.

## 2 Case presentation

A 22-year-old Iranian male presented to the Emergency Room with a sudden onset of chest pain and dyspnea, without any significant past medical history. He was a construction laborer, and his symptoms began abruptly while lifting a heavy object.

**Fig. 1:**
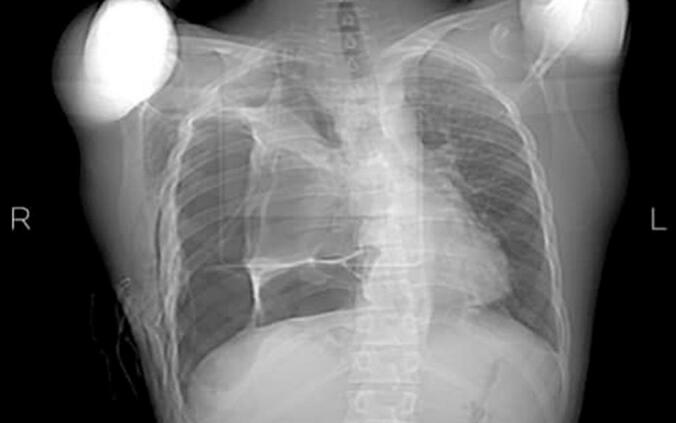
CXR in the first treatment center.

Based on the symptoms, physical examination and chest X-ray findings, the patient was diagnosed with pneumothorax at the referring hospital, where a chest tube was inserted into his right hemithorax (Fig. 1). However, as his symptoms persisted, the lung failed to expand properly, and no underlying cause of the pneumothorax was identified. Due to the lack of progress, our surgical team was consulted, and the patient was transferred for further evaluation.

Given our experience with patients presenting with pulmonary symptoms in endemic areas who were later diagnosed with hydatid cysts, this differential diagnosis was considered. Subsequent investigations and CT scan imaging revealed the characteristic “water lily sign,” confirming the diagnosis.

**Fig. 2:**
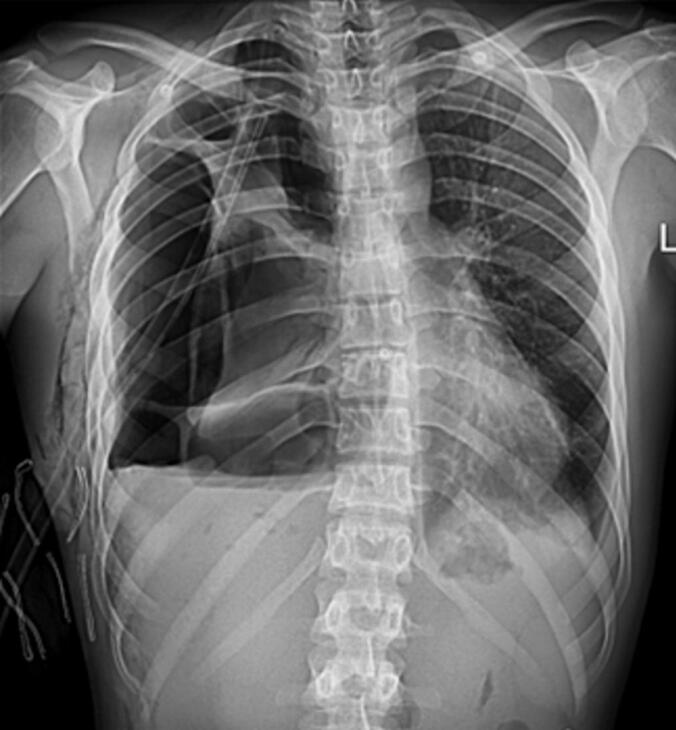
CXR before operation (pneumothorax and chest tube can be seen).

Despite the presence of a chest tube, the right lung remained completely collapsed due to infection and necrotic lesions filling the pleural cavity (Fig. 2). Notably, the patient exhibited no fever, tachypnea, or other signs of respiratory distress. His initial white blood cell count was approximately 14,000, while other laboratory parameters remained within the normal range.

Surgical intervention was the choice for removing the cyst and surrounding tissues. Therefore, the patient was prepared for thoracotomy and an anterolateral incision was performed. Upon opening the chest wall, a massive of empyema was observed within the pleural cavity and the infection was widespread. Remnants of the laminated membrane of the cyst were located in the right middle lobe. All necrotic tissue and cyst membranes were excised as thoroughly as possible. Given the presence of multiple broncho-pleural fistulas, indicated by persistent chest tube bubbling, we meticulously occluded the fistulas and repaired them using PDS sutures. to ensure no residual air leaks, we instilled normal saline into the pleural cavity and reinflated the lung; fortunately, no leakage was detected. A new chest tube was placed, and the chest wall was repaired routinely.

**Fig. 3:**
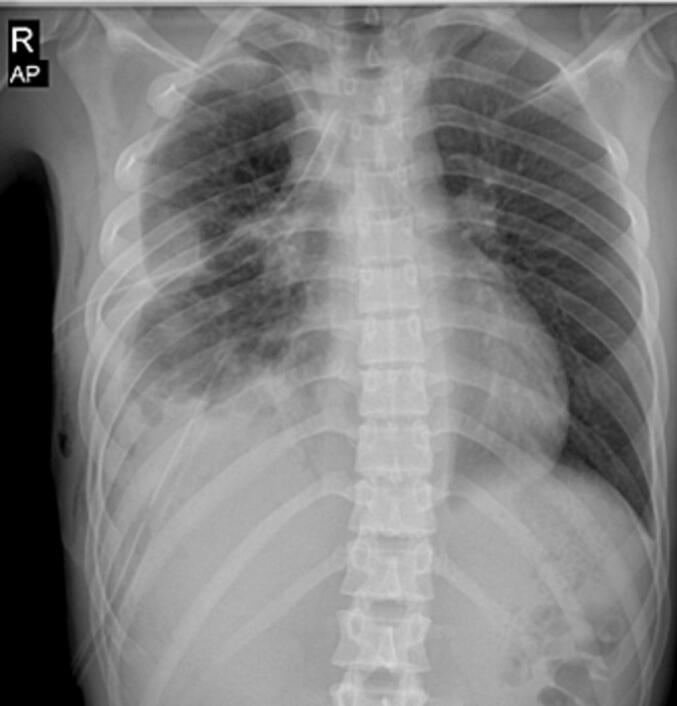
CXR four days after surgery and after insertion of the second chest tube.

**Fig. 4:**
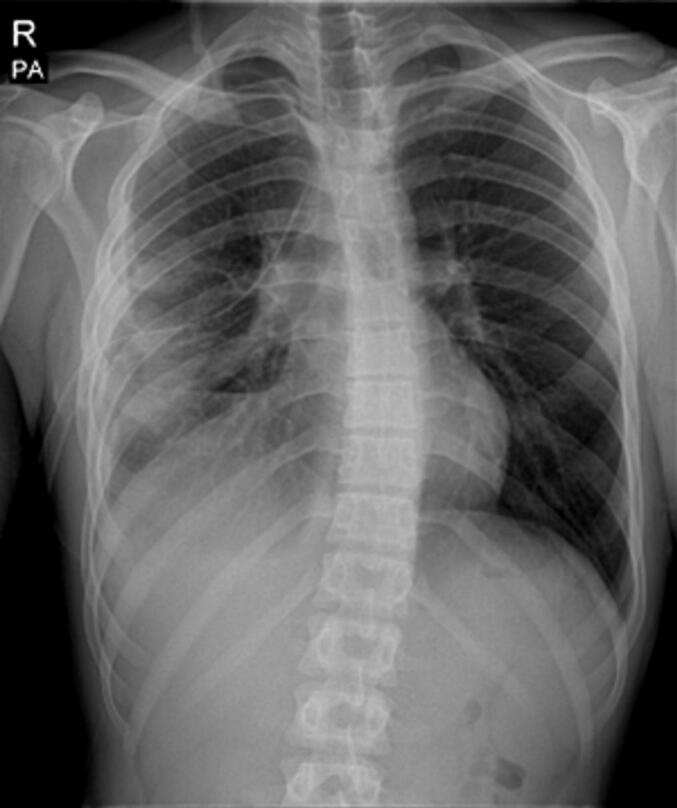
CXR eighteen days after surgery.

On the fourth day post-operation, the patient's pulmonary symptoms had nearly resolved. To provide a better chest expansion, we inserted an additional chest tube in the upper intercostal area (Fig. 3). The patient was started on intravenous broad-spectrum antibiotics, including Tazocin and Meropenem, to manage the secondary bacterial infection and prevent further complications. This antibiotic regimen led to marked clinical improvement, with the patient's WBC count decreasing from 17,000 on the first postoperative day to 6000 by the seventh day. Antibiotic therapy continued throughout the hospital stay and was followed by an oral regimen upon discharge. Ultimately, after seven days in the surgical ward, he was discharged in good condition with the chest tube connected to a urine bag. A week later during a follow-up examination, we removed the chest tube and no further complications were detected. Fig. 4 shows the follow-up CXR eighteen days after surgery. The work has been reported in line with the SCARE criteria. [9]

## 3 Discussion

Hydatid disease is classified as a neglected tropical disease and is rare in many parts of the world; however, in endemic areas, including sub-Saharan Africa and the Middle East, it remains a significant public health threat [7]. Lungs are the second most common site of cyst formation, after the liver, and clinical symptoms vary depending on the size and condition of the cyst. if a cyst ruptures, it can lead to complications such as pleural effusion and empyema [10].

In most cases, symptoms remain silent and the cyst grows unnoticed until incidental imaging or pressure on adjacent organs reveals their presence [11].Ruptures can be classified as contained or complete. In a contained rupture, the pericyst detaches from the endocyst while remaining intact, without perforation. in a complete rupture the cyst establishes a connection with the bronchial tree or, less commonly, the pleural cavity [10]. In these scenarios, pneumothorax and empyema are among the rare complications that may occur in less than 5 % and 7 % of the cases respectively [12]. Although parasitic infections are among the least likely differential diagnoses for empyema, as demonstrated in this case, hydatid disease can cause fulminant empyema and create a fistula between the pleural cavity and the bronchial tree.

In the case of non-complicated cysts, a chest X-ray is proven to be a beneficial diagnostic tool; however, for complicated cysts, a CT scan is the preferred imaging modality. Laboratory findings are usually nonspecific although an isolated rise in white blood cell count is probable [10]. In our case, the presence of the characteristic “water lily sign” on CT imaging aided in confirming the diagnosis.

Surgical intervention is the preferred treatment, particularly in patients with complicated or ruptured cysts. According to the size and condition of the cyst, different surgical approaches can be taken; if the cyst remains intact, needle aspiration or enucleation may be performed. However, in cases of rupture, such as the one presented, the remnants of the cyst, along with empyema and necrotic lung tissue, must be excised. in both cases, thoracotomy provides easier access for the complete removal. Additionally, all pleuro-bronchial fistulas should be completely closed with sutures to prevent further complications [13,14]. A combination of surgery and Albendazole therapy has been shown to improve outcomes. In cases where the cyst remains intact or the patient is not a surgical candidate, Albendazole alone serves as the optimal treatment [15].

## 4 Conclusion

In endemic regions, hydatid cysts should be considered in the differential diagnosis of nonspecific pulmonary symptoms, particularly when standard treatments fail. Spontaneous rupture can rapidly lead to complications such as empyema, broncho-pleural fistulas, and fibrotic tissue formation. Early diagnosis using CT imaging and prompt surgical excision of cyst remnants and necrotic tissue are essential for optimal outcomes. Combining surgery with targeted antibiotic therapy significantly reduces morbidity and improves recovery in these patients.

## CRediT authorship contribution statement

MB and VA developed study principles and design. VA and PS wrote the manuscript. MB and AA revised and confirmed the article. All authors approved the submitted version of the article.

## Patient consent

Complete written informed consent was obtained from the patient for the publication of this study and accompanying images.

## Ethical approval

The ethical approval was exempt. We did not obtain an approval reference number at the time of preparing this case report, as it was conducted in accordance with our institution's policies which do not require formal ethical approval for single-patient case reports with informed consent.

## Guarantor

Vahid Ahmadi.

## Funding

The authors of this paper receive no funding from any organization or research foundation.

## Declaration of competing interest

The authors declare that they have no known competing financial interests or personal relationships.

## Data Availability

The datasets generated during and analyzed during the current study are available from the corresponding author upon reasonable request.
